# Epidemiology, Clinical Features, and Prescribing Patterns of Aortic Aneurysm in Asian Population From 2005 to 2011

**DOI:** 10.1097/MD.0000000000001716

**Published:** 2015-10-16

**Authors:** Shih-Wei Wang, Yaw-Bin Huang, Jiann-Woei Huang, Chaw-Chi Chiu, Wen-Ter Lai, Chung-Yu Chen

**Affiliations:** From the School of Pharmacy, Master Program in Clinical Pharmacy (S-WW, Y-BH, C-YC); Department of Pharmacy (Y-BH, C-YC); Department of Surgery, Division of Cardiovascular surgery (J-WH, C-CC); and Department of Internal Medicine, Kaohsiung Medical University Hospital, Kaohsiung, Taiwan (W-TL).

## Abstract

Aortic aneurysm (AA) is a leading cause of death in Asia and the world. The prevalence in Western countries is around 1.3% to 8%. However, it is still unclear about the incidence, prevalence, and mortality of AA in Asian population. The aim of this study is to investigate the epidemiology of AA for all subtypes in Taiwan, and describe the clinical features and prescribing patterns for AA population.

A population-based study was conducted using information from National Health Insurance Research Database (NHIRD) in Taiwan. Patients who were diagnosed with AA and also received computed tomography (CT) were included in this study. Incidence, prevalence, and mortality were calculated in each year during 2005 to 2011. Prevalent comorbidities and prescribing patterns were both evaluated among study population.

The average annual incidence of AA in Taiwan was 7.35 per 100,000 population, and the prevalence was 29.04 per 100,000 population. It showed an increased trend of incidence from 2005 to 2011, so as prevalence and mortality. The incidence was associated with age and sex difference. It was much higher in those older than 65 years, especially for male. Hypertension, coronary artery disease (CAD), and chronic obstructive pulmonary disease (COPD) were prevalent comorbidities. Eighty-eight percentages of patients were prescribed antihypertensive agents in acute phase, where 61.4% of calcium channel blocker (CCB) was the most one.

Our study found that incidence of AA was lower in Taiwan than in other countries. Nevertheless, it showed an increased trend of AA disease for incidence, prevalence, and also mortality during 2005 to 2011.

## INTRODUCTION

Aortic aneurysm (AA) is a leading cause of death in Asia and the world, and usually presents asymptomatic until ruptures, leading to high mortality. The prevalence of abdominal aortic aneurysm (AAA) reported in Western countries is around 1.3% to 8%.^1–5^ However, there are rare epidemiological reports among Asian population. Only few studies from Japan,^6^ Hong Kong,^7^ and Korea,^8,9^ and the prevalence in Asia shown to be much lower than in Western countries. Most of these prevalence data are from the results of AAA screening programs, and using detection rate as prevalence rate. It is still unclear about the incidence, prevalence, and outcome of AA for all subtypes in general Asian population.

Numerous studies have shown the risk factors of AA including tobacco use,^8,10–13^ coronary artery disease,^11,13,14^ hypertension,^8,10,13^ and hyperlipidemia.^10,15^ As per the result from a meta-analysis, the risk factors varied from region to region.^16^ Limited data were established for the feature of patients with AA disease in Asia. Furthermore, the effectiveness of medical therapy for AA remained controversial. Choices of medication for AA in Taiwan were based on physicians’ experiences. However, no nationwide research focusing on AA prescribing patterns in Taiwan has been published.

The objective of this study is to investigate the epidemiology of AA in Taiwan. In addition, the nationwide study aims to describe AA disease by subtype, sex, age, comorbidities, and outcome, as well as to evaluate utilization of medication therapy.

## MATERIAL AND METHOD

### Data Sources

We conducted a population-based study using information from the National Health Insurance Research Database (NHIRD) in Taiwan. Whole population in Taiwan with at least 1 diagnosis of AA during 2004 to 2012 would be included in this database. Detail information including outpatient care, inpatient care, emergency care, and prescription were provided by National Health Insurance (NHI) Research Institutes for use in this study. The NHI program was established since 1995 and provided coverage for 99.6% of 23,162 million residents in Taiwan by 2012.^17^ The annual numbers of total population in Taiwan was quoted from national reports published by Department of Household Registration Affairs, Ministry of the Interior. This study was approved by the Institutional Review Board of Kaohsiung Medical University Hospital on December 25, 2014.

### Patient Definition

We identified patients who were diagnosed as having aortic aneurysm (International Classification of Diseases, ninth revision, Clinical Modification [ICD-9-CM]: 441.1–441.7, 441.9) between January 1, 2004 and December 31, 2012. Since computed tomography (CT) was generally considered the criterion standard for diagnosis of AA,^18^ we excluded patients without CT scan within 1 year after index date. The index date was assigned as the first date of AA diagnosis.

### Comorbidity, Prescribing Pattern, and Outcome

Comorbidities were extracted from ambulatory visit and hospitalization data within a year before index date by ICD-9-CM code. Comorbid disease was defined with at least 2 ambulatory or 1 hospitalization diagnoses. Diseases included hypertension, dyslipidemia, heart failure, diabetes mellitus, chronic obstructive pulmonary disease (COPD), coronary artery disease (CAD), cerebrovascular disease (CVD), peripheral vascular disease, chronic kidney disease, atherosclerosis, arrhythmia, gout, thyroid disease, transplant, and any cancer. Medication evaluation included all antihypertensive agents (including angiotensin-converting enzyme inhibitor [ACEI], angiotensin II receptor blocker [ARB], calcium channel blocker [CCB], β-blocker, diuretics, α-blocker and vasodilator), antiplatelet agents (including aspirin, clopidogrel, dipyridamole, and cilostazol), lipid-lowering agents (HMG-CoA reductase inhibitor [statin]) and other medication associated with comorbidities. User was defined as exposure over 1 month in each period. Outcome measurements included receive operation and death during study period. Operated status was defined by using ICD-9-CM procedure code (open repair [OPR]: 38.44, 38.45; endovascular repair [EVAR]: 39.71, 39.73).

### Statistical Analysis

Annual incidence and prevalence of AA were calculated as the number of incident or prevalent cases divided by the number of total Taiwanese population in each year. Mortality and case fatality rate were also computed in each year. Rates were presented as number of cases per 100,000 population, except case fatality rate. Descriptive statistics were presented as total number of cases and proportion, including items of characteristics, comorbidities, outcome, and prescribing patterns. All of the analyses in this study were performed using SAS version 9.3 (SAS Inc, Cary, NC)

## RESULT

**FIGURE 1:**
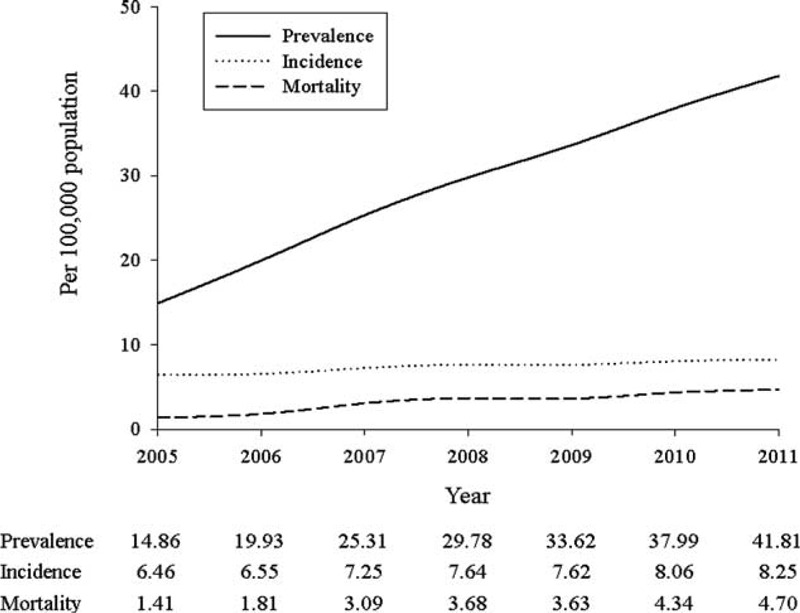
Incidence, prevalence, and mortality of AA population in Taiwan.

A total of 11,939 patients were identified with AA during 2005 to 2011 in Taiwan. The average incidence was 7.35 patients with newly diagnostic AA per 100,000 population. Figure 1 shows the trends of incidence, prevalence, and mortality of AA in Taiwanese population. Incidence was 6.46 per 100,000 population in 2005 and increased by almost 28% from 2005 to 2011. The highest incidence was 8.25 per 100,000 population in 2011. Incidence in male was 2 times higher than in female. The prevalence of AA was a rising trend from 14.86 to 41.81 per 100,000 population during 2005 to 2011. The prevalence in male was 3 times as much as the prevalence in female. Mortality was 1.41 per 100,000 population in 2005 and increased as high as 4.7 per 100,000 population in 2011. Case fatality rate was also calculated and the range was around 9.49% to 11.25% during 2005 to 2011 (Table 1).

**TABLE 1:**
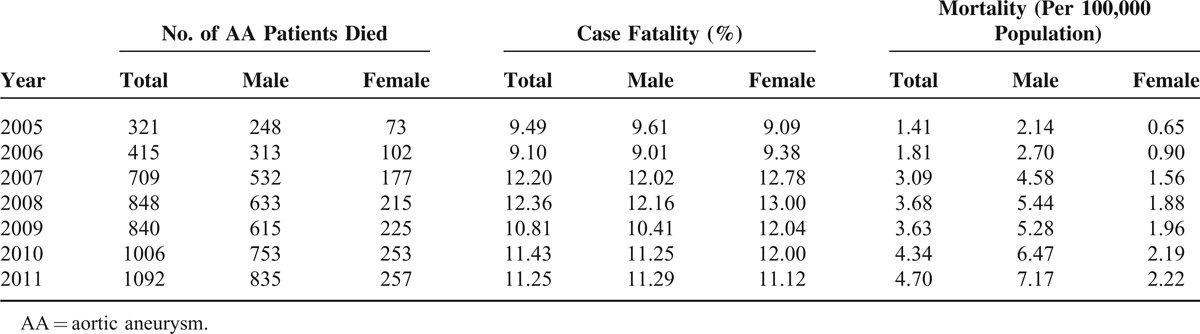
Mortality and Case Fatality of AA Population During 2005–2011 in Taiwan

Baseline characteristics of AA population were presented in Table 2. Among 11,939 patients included in this study, 9412 (78.83%) were elderly and 9022 (75.6%) were male. The mean age was 73.3 years. AAA was the major subtype and accounted for 48.5%. Thirty-three percentages of patients have received operation during our study period. A total of 4128 surgeries were done for 4042 patients, wherein 2615 (63.3%) cases underwent OPR and 1503 (37.7%) cases underwent EVAR. A total of 5218 (43.7%) patents died during the follow-up time.

**TABLE 2:**
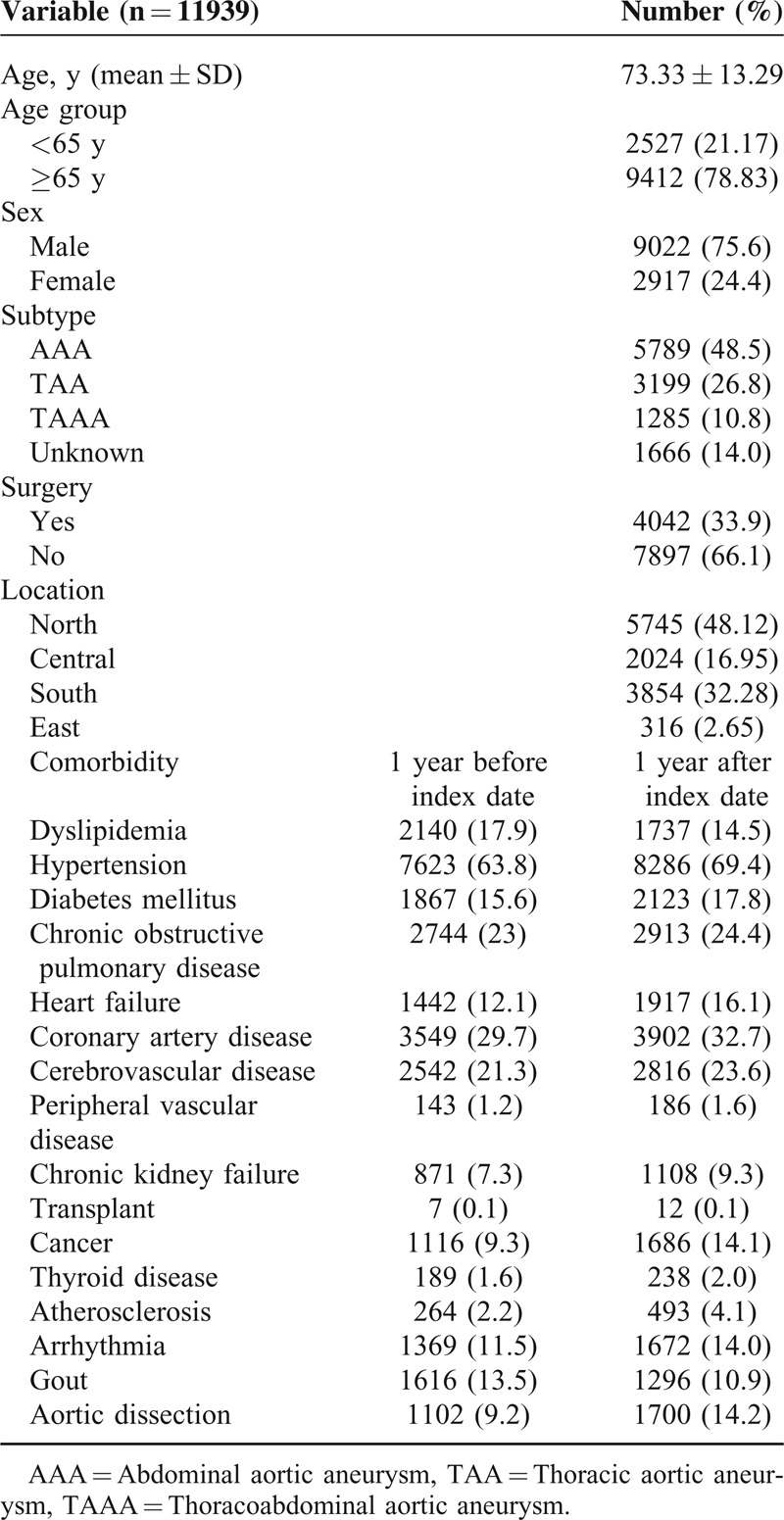
Baseline Characteristic of AA Population in Taiwan

Prevalent comorbidities included hypertension (63.8%), CAD (29.7%), COPD (23%), and CVD (21.3%) within 1 year before index date. These comorbidities remained prevalent at 1-year period after index date. Furthermore, the rates of these comorbidities were higher in the period after index date, except dyslipidemia and gout.

To address potential prescribing patterns changed over time, the evaluation was divided into several phases (Table 3). The prescribing rate of antihypertensive agents was the highest one among all periods; of these, CCB was the major agent. The prescribing rate of antihypertensive agents was 75.1% in the baseline period, and increased to 81.4%, 85.5%, and 88.7% in 3 months, 6 months, and 1 year after index date, respectively. The prescribing rates of antiplatelet agents in different periods were fluctuated. It showed 39.7% in baseline, dropped to 37%, and then increased to 47.2% after 1 year after index date. The prescribing rate of aspirin was the highest one among the class of antiplatelet agents. Sixteen percent of patients in baseline period had been prescribed statin and the rate was increased to 19.1% after 1 year. Table 3 shows the prescribing rates of each class of medication.

**TABLE 3:**
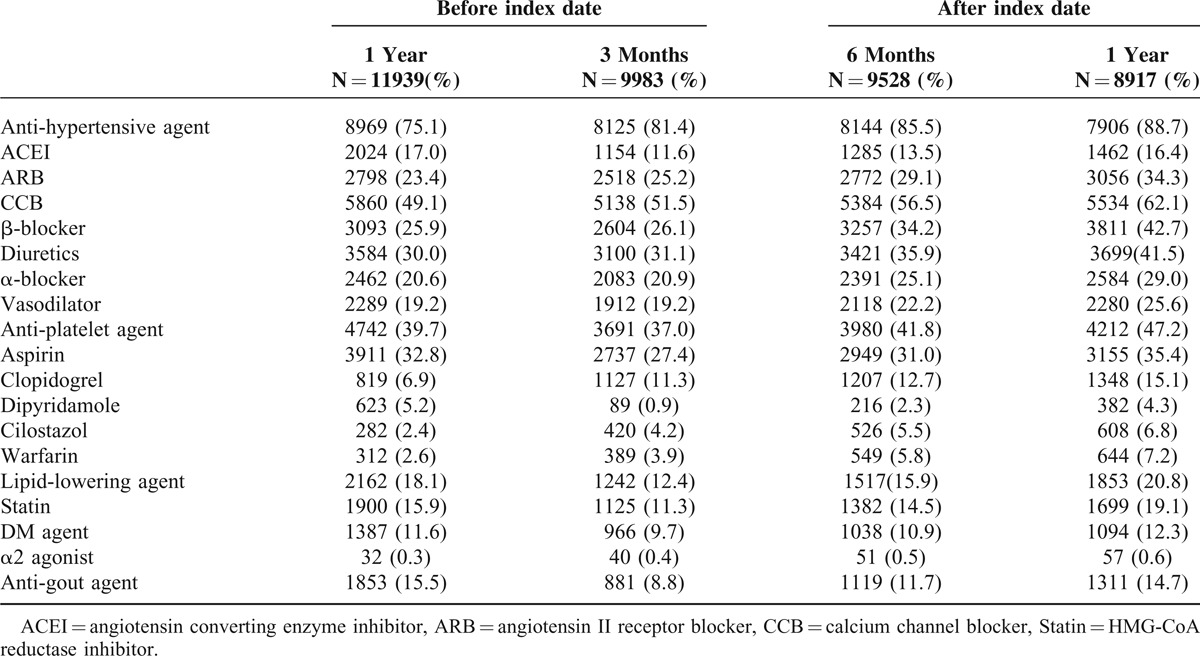
Prescribing Pattern of AA Population in Taiwan

## DISCUSSION

**FIGURE 2:**
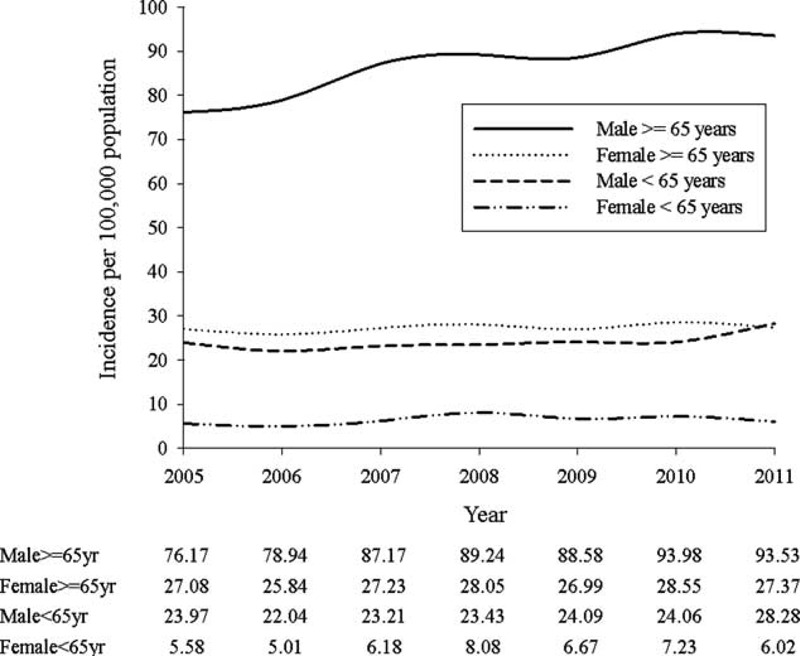
Incidence of AA population in subgroup of sex and elderly.

The population-based epidemiological study examined the incidence, prevalence, and clinical characteristics of AA patients among patients with all subtypes over a period of 7 years. The major strength of our study is that we reported population-based epidemiological data and prescribing patterns in Asian population. The results revealed that the average incidence of AA was calculated as 7.4 per 100,000 population, and almost 80% cases of newly diagnostic AA were borne by those aged above 65. The incidence was strongly associated with age. The average incidence among elderly was 56.1 per 100,000 population; also, it was higher than the average incidence (7.4 per 100,000 population) in general population. The incidence was also related to sex difference. The incidence in men and women were 11.09 and 3.65 per 100,000 population, respectively. After all, the incidence increased rapidly after the age of 65 years, especially for male (Figure 2).

Comparing with epidemiological studies in Western countries, our study showed a relatively low incidence among Taiwanese population, which was consistent with previous studies from Asian countries. In Hong Kong, Cheng et al^7^ showed that the annual incidence of AAA was 13.7 per 100,000 and was estimated to be 105 per 100,000 for those older than 65 years. Yii et al^19^ also reported that the incidence for males older than 50 years was 25.6 per 100,000, and was 78.3 per 100,000 for males older than 70 years in Malaysia. However, the incident rate in our study was lower than reported rates among other Asian countries.

The trend of incidence was increasing from 2005 to 2011; it might be attributed to the increasing screening rate of CT. The aging of Taiwanese population might be another reason. Old population increased from 2,216,804 to 2,528,249 in Taiwan during the study period. Since formation and enlargement of AA were considered to be associated with age, the incidence will get higher in the following time while there are more elderly in our population.

On the contrary, AA mortality increased during these years. One study^20^ estimated the global AA death rate and showed that it was 2.49 in 1990 and 2.78 per 100,000 population in 2010. The trend in Asia also increased between 1990 and 2010. On the contrary, in Australasia, Western Europe, and North America, which were having the highest mortality of AA among the global burden of disease regions in 1990, AA mortality rates were all in a declined trend. Our finding was consistent with the trend of AA mortality in Asian population.

The prevalence of AA in Taiwan was much lower than in other screening data (Table 4). Adachi et al^6^ showed that the prevalence of those older than 65 years was 0.3% in Japan. Scott et al^1^ found that the prevalence in the group of men aged 65 to 74 years was 4.9% in UK. In our result, it showed 0.05% in the elderly population. Although the prevalence was much lower than previous results, it is unequal to compare our local epidemiological data with other screening data. The prevalence calculated from database might be lower than previous data from the reports of screening programs.

**TABLE 4:**
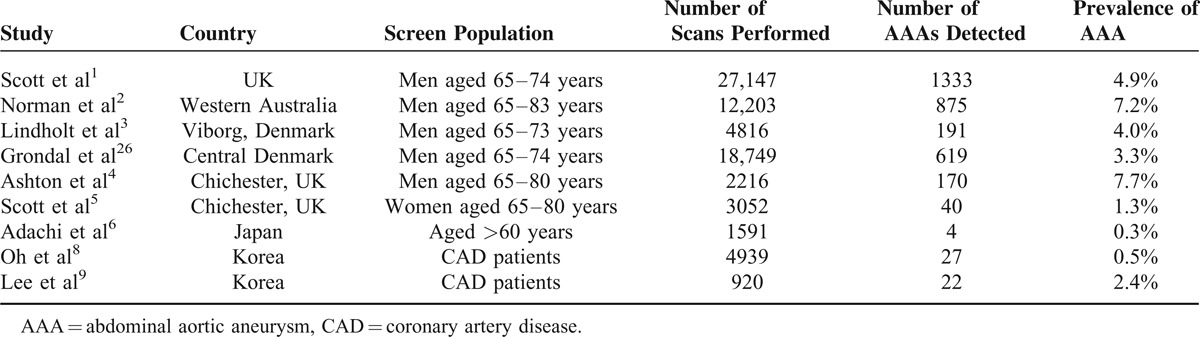
Prevalence of AAA in Screened Population

We found that both incidence and prevalence were relatively low in Taiwanese population. The possible reason of the phenomena might be that there is no routine CT screening program in Taiwan, and asymptomatic AA is an imperceptible disease for general population. Owing to that, only symptomatic AA patients and those who underwent other image examination (eg, ultrasound) for other diseases or physical examination might be suspected with AA and arranged for further CT scan to confirm the diagnosis. Those without symptom and any previous image examination would not seek medical advice.

A rapid decline trend was shown in Table 3. It declined from 11,939 patients at index date to 8917 at 1 year later. The reasons for the rapid decline were as following. First, the life expectancy of Taiwanese population was around 78 years and the median age in our study population was 76.42 years. Since AA patients were older and with worse condition than healthy population at the same age, the survival time might be shorter than average. Second, it might be partially attributed to patients who underwent AA-related surgery as well as critical patients with ruptured AA. The mortality was high while aneurysm ruptured or patients underwent surgery.^21,22^

The risk factors of AA such as hypertension and CAD were consistently prevalent in our population. Besides, COPD was also one of common comorbidities in our study population. From a previous study, Flessenkaemper et al found that patients with COPD were predominately current or former smokers.^23^ Although smoking status was not available in our database, COPD morbidity rates could be used as a surrogate to prove the association between tobacco use and AA in our study.

According to guideline,^24^ controlling blood pressure is important for AA population. It is consistent in our finding that prescribing rate of antihypertensive agents after index date was higher than during baseline period. To evaluate the initial treatment of AA, exposure rates of each drug within 30 days after index date were estimated. In acute phase, prescribing rate of antihypertensive agents was 87.5%, wherein 61.4% of CCB was the highest one, and the following were β-blocker (49.3%), diuretics (41.4%), and vasodilators (30.8%). Clinical guideline recommended that β-blocker should be administered to all patients with AA to reduce the growth rate of aneurysms. ACEI and ARB were also reasonable to be administered for reducing the blood pressure. However, our data showed that the prescribing rate was not as high as expectated. It seems that physicians in Taiwan may not always follow the guideline to treat these AA patients in acute phase.

For dyslipidemia, statin was recommended for those who coexisted with coronary heart disease. In our study, the proportion of statin user was relatively lower than that in a previous hospital-based study,^25^ which is 54% (349/652). One of the reasons might be that the comorbidity rate of dyslipidemia was low (17.9% in baseline; 14.5% after index date) in our study population. Physicians in Taiwan might be more conservative than in Western countries. Statin would only be prescribed when patients really coexisted with dyslipidemia. Another reason might be that if low-density lipoprotein was in a normal range, the NHI would not cover the drug price, and patients needed to pay by themselves. Since self-paid prescriptions would not be available in NHIRD, so the prescribing rate of statin might be underestimated.

Nowadays, the effectiveness of aspirin for preventing aneurysm growth and reducing mortality remains controversial. Some of previous studies showed that aspirin did not decrease the growth rate of aortic aneurysms significantly.^25–27^ However, Lindholt et al^28^ indicated that low-dose aspirin may prevent the expansion rate for medium-size AAA (<40 mm). In our study, aspirin use was decreased in the acute phase (29.3% vs 32.8% at baseline). The decreased trend could be explained by the bleeding risk of aspirin. Considering the bleeding risk, physician may not prescribe aspirin unless for other needed. Clopidogrel, with minor bleeding risk, may be another choice instead of aspirin for those who had indication for antiplatelet. So, the prescribing rate of clopidogrel increased in the acute phase (12.8%).

This study is the first epidemiological study of AA in Taiwan. No previous population-based epidemiological study described AA among all subtypes with such a long period. All studies in Western countries or even in Asia were based on screening examination, which was launched for men older than 65 years or for population with specific disease (eg, CAD).

National screening programs have already been initiated for men older than 65 years in England, Scotland, Sweden, and the United States, but not executed in Taiwan. However, elderly population is increasing here and as high at 11.3% by 2013. This study suggests that routine screening program should be established for men older than 65 years or population in a high risk for AA. In addition, the study provides a greater understanding of the epidemiology, clinical features, and outcomes of AA at a population level, which may inform future screening program strategy.

Nevertheless, there are still several limitations in our study. First, without image data is the main limitation of this database. CT scan is the criterion standard to diagnose AA and provides the diameter of aorta, which could determine the severity and associated with the rupture risk as well as survival outcome. There was no medical imaging record in this database, which could be used to confirm the diagnostic accuracy. Therefore, we excluded patients without receiving CT scan within 1 year after index date or having only once AA diagnostic code during follow-up time to confirm the diagnostic accuracy in our database. However, the low estimated number of cases in this study may cause by some AA patients were excluded due to without receiving CT examination within 1 year after index date or they only had once AA diagnostic code in our survey. Second, smoking status was not available in the database. The direct association between tobacco use and AA would not be established in our study. Third, since the duration of database, we may lose a number of patients who had AA diagnosis before 2004 and still alive, but lost to follow-up during 2004 to 2011. It may lead to the underestimation of both the prevalence and incidence. Fourth, AA is an asymptomatic disease until aneurysm ruptures, and it is imperceptible for general population. For those, who may have small AA but without any symptom and without further diagnosis, would not have medical record in NHIRD. Hence, it may be underestimated the true prevalence and incidence in Taiwan. Further screening programs may be needed to confirm the epidemiological data in our study.

## CONCLUSIONS

The incidence of AA in Taiwan was lower than in Western countries, and even in other Asian countries. Although the incidence might be underestimated in our study due to the limitation of database, the burden of aortic aneurysm was increased in Taiwan, especially for elderly population. The characteristics of AA population in our findings were similar to previous studies. Hypertension, CAD, and COPD were the top prevalent comorbidities in our population. In acute phase of AA, CCB, β-blocker, and vasodilator were the choices for initial treatment. However, the prescribing rates of medication for AA in Taiwan were lower than those reported in previous studies. In the future, more studies are needed for further investigating AA disease.
